# The Bioavailability and Pharmacokinetics of Silymarin SMEDDS Formulation Study in Healthy Thai Volunteers

**DOI:** 10.1155/2018/1507834

**Published:** 2018-07-19

**Authors:** Chuleegone Sornsuvit, Darunee Hongwiset, Songwut Yotsawimonwat, Manatchaya Toonkum, Satawat Thongsawat, Wandee Taesotikul

**Affiliations:** ^1^Pharmacy Service Center, Pharmacy Faculty, Chiang Mai University, Chiang Mai 50200, Thailand; ^2^Department of Pharmaceutical Care, Chiang Mai University, Chiang Mai 50200, Thailand; ^3^Department of Pharmaceutical Sciences, Chiang Mai University, Chiang Mai 50200, Thailand; ^4^Department of Internal Medicine, Faculty of Medicine, Chiang Mai University, Chiang Mai 50200, Thailand

## Abstract

The present study aimed to determine the pharmacokinetic parameters and bioavailability of silymarin 140 mg SMEDDS formulation. An open-label, single-dose pharmacokinetic study was conducted. Twelve healthy volunteers were included in the study. After the volunteers had fasted overnight for 10 h, a single-dose generic silymarin 140 mg SMEDDS soft capsule was administered. Then 10 ml blood samples were taken at 0.0, 0.25, 0.50, 0.75, 1.0, 1.33, 1.67, 2.0, 2.5, 3.0, 4.0, 6.0, 8.0, 10.0, and 12.0 h. The plasma silybin concentrations were analyzed using validated LC-MS/MS. The pharmacokinetic parameters were analyzed and calculated. The pharmacokinetic parameters were calculated after silymarin had been administered as a single capsule. The mean (range) C_max_ was 812.43 (259.47–1505.47) ng/ml at 0.80 (0.25–1.67) h (t_max_). The mean (range) AUC_0-t_ and AUC_0-inf_ were 658.80 (268.29–1045.01) ng.h/ml and 676.98 (274.10–1050.96) ng.h/ml, respectively. The mean k_e_ and t_1/2_ were 0.5386 h^−1^ and 1.91 h, respectively. The silymarin SMEDDS formulation soft capsule showed rapid absorption and high oral bioavailability.

## 1. Introduction

Silymarin, a flavonoid found in the seeds and fruit of milk thistle extraction (*Silybum marianum* or* Carduus marianus*) has been used for centuries as a treatment for liver-related disease [1, 2]. The plant grows in Europe, North America, South America, Africa, Australia, India, and China. Silymarin consists of silybin A and B (the primary and most active component), isosilybin A and B, silydianin, and silychristin. Silymarin has a hepatoprotective effect with various mechanisms of actions including, as antioxidant, scavenging and being involved in glutathione antioxidant functions; as biomembrane stabilizer and regulator, resulting in prevention of toxic substance passing into hepatocytes; as protein synthesis stimulator, resulting in liver regeneration; and as inhibitor of stellate hepatocyte, the change to myofibroblasts, which is the process of the deposition of collagen fibers leading to cirrhosis. Moreover, silymarin also has anti-inflammatory and anticarcinogenic effects [1–3]. These properties could lead silymarin to potential use in the treatment of numerous liver diseases such as acute and chronic viral hepatitis, toxin- or drug-induced hepatitis, cirrhosis, and alcoholic liver disorders. It has also been effective in the treatment of certain types of cancers, e.g., breast, prostate, and skin cancers [2, 4]. Canada has approved around 70 different products containing this herb. It makes approximately 180 million US dollars per year in Germany [5].

The effectiveness of silymarin diminishes in its use as a hepatic medication due to its poor solubility in water and its low oral bioavailability [6]. Silybin shows only 20–50% absorption by the gastrointestinal tract. Silybin has an absolute oral bioavailability of 0.95% [3, 7, 8]. This may be a result of its high reactivity with phase II conjugation, poor permeability through the epithelial cells in the gut, poor water solubility, and rapid elimination via bile and urine [6]. Silybin undergoes extensive enterohepatic circulation. 18% of absorbed silybin is excreted in the bile after conjugation with glucuronide and sulfate. Only a small amount is excreted in the urine. Silybin concentrations in bile are 60 times higher than those in plasma. Many methods have been developed and investigated to improve the dissolution and bioavailability of silymarin, including formation into a complex, incorporation into solid/semisolid dispersion, encapsulation in liposome, and solubilization in a self-microemulsifying drug delivery system (SMEDDS) [6, 9–12]. Out of these, the SMEDDS has attracted huge attention in the pharmaceutical industry. In addition, its high thermodynamic and kinetic stability, low viscosity, and optical transparency make it very attractive for pharmaceutical applications. The SMEDDS is a mixture of poor water-soluble drugs, lipids, surfactants, and cosurfactants, which forms a fine oil-in-water microemulsion (droplets smaller than 100 nanometers) when dispersed in watery media with gentle agitation or in digestive fluids with movements of the gut. The enhancing absorption mechanisms increase the interfacial surface area, resulting in the formation of a dissolved drug microemulsion ready for release and absorption. Furthermore, the unique compositions of the SMEDDS increase membrane fluidity resulting in the easy transcellular passing, open tight junction facilitating para-cellular transport, inhibit P-gp and/or CYP450 resulting in increment of intracellular available and residual time by surfactants, and stimulate lipoprotein/chylomicron production of fat [7, 13].

A few studies have formulated silymarin in a SMEDDS in order to improve the solubility, dissolution, and absorption of silymarin. Pharmacokinetic studies on silymarin SMEDDS formulation have only been performed on animals (rats, rabbits, and dogs) [7, 13, 14]. However, there are insufficient data concerning the study of pharmacokinetics in humans. Therefore, this study was conducted in order to investigate the pharmacokinetic parameters of silymarin SMEDDS formulation in healthy subjects in fasting conditions.

## 2. Materials and Methods

### 2.1. Volunteers and Study Design

Twelve healthy male and female volunteers, aged between 18 and 45 years old with a body mass index between 18.5 and 25.0 kg/m^2^, were recruited for an open-label, single-dose under fasting conditions pharmacokinetics study. The inclusion criteria were that the volunteers had to be healthy according to their medical history, they had to undergo a physical examination, they had to be hepatitis B negative according to their clinical laboratory test results, they had to be nonsmoking, females had to be nonpregnant who were either unable to have children or committed to using acceptable nonhormonal contraceptives if potentially childbearing, and finally they had to be willing to comply with the study's procedures and restriction. Volunteers not included were those with a history of allergy to silymarin, a related structure of silymarin, or other components in the formulation; those who had a current or history of alcohol addiction or drug abuse; those who had been using any medication including vitamins, herbal products, and dietary supplements up to 14 days before or during the study; those who had consumed oranges, pomelos, or grapefruits up to 7 days before or during the study; and finally those who had ingested caffeine-containing beverages or food up to 3 days before or during the study. Any volunteers who had donated more than 300 ml of blood, suffered significant blood loss, or participated in other clinical trials up to 90 days before the start of the study were also excluded. All the volunteers were briefed on the details of the study. The volunteers who met the above criteria were eligible for participation after voluntarily signing their informed consent.

The study was carried out at the Clinical Trial Unit (CTU) at the Faculty of Medicine at Chiang Mai University in Chiang Mai, Thailand, and complied with the International Conference on Harmonisation's Good Clinical Practice Guidelines. The Research Ethics Committee at the Faculty of Medicine at Chiang Mai University, Chiang Mai, Thailand, approved the study's protocol on 21st January 2016.

### 2.2. Silymarin Product, Administration, and Blood Sample Collection

The silymarin SMEDDS formulation used in this study comprised Silyvercell® Soft Capsules manufactured by Phil Inter Pharma Co. Ltd. in Vietnam, which were made of 175 mg* Carduus marianus* extract, equivalent to 140 mg silymarin, and calculated as 60 mg silybin. The SMEDDS was prepared using a mixture of* Carduus marianus* extract, polysorbate 80, polyoxyl-40-hydrogenated castor oil, polyethylene glycol 400, and propylene glycol. The recommendation dose of silymarin is a range from 70 to 140 milligrams three time per day in various disease conditions; thus one capsule of 140 mg silymarin was used for pharmacokinetic study.

Every volunteer who had fasted for at least 10 h and stayed at the CTU the night before the administration received a single oral dose silymarin SMEDDS capsule with 240 ml drinking water. Drinking water up to 1 h before or after taking the medication was not allowed. A standardized meal for lunch and dinner was served 4 and 10 h after the dose.

Ten ml blood samples were collected from each volunteer using a blood collection tube coated with K_2_EDTA 0.0, 0.25, 0.50, 0.75, 1.0, 1.33, 1.67, 2.0, 2.5, 3.0, 4.0, 6.0, 8.0, 10.0, and 12.0 h after the silymarin administration. They were then transported at a controlled temperature to the Pharmacy Service Center at the Pharmacy Faculty at Chiang Mai University, where the plasma samples were immediately separated and stored at −30 ± 5°C until their analysis.

### 2.3. Analysis of Silybin in Plasma

An analytical method for measuring silybin in human plasma by LC-MS/MS was developed. The analytical procedures are briefly described as follows.

Sample Preparation. Thirty microliters of 7 *μ*g/ml working solution of NRG (internal standard) was subsequently added to 750 *μ*l of plasma prepared for calibration standards, QC samples, and study samples. After adding 6 ml ethyl acetate, the mixture was mixed using Vibramax 110 shaker at 1500 rpm for 15 min at room temperature and then centrifuged using a Hermle Z 383K at 3000 rpm for 1 min at 20°C. A 4 ml portion of organic phase was collected, transferred into another test tube, and evaporated to dryness under a stream of 5 psi nitrogen gas for 40 min at 25°C. The dried residue was dissolved with 200 *μ*l of mobile phase and shaken using a Vibramax 110 shaker at 2000 rpm for 5 min at room temperature. An aliquot was centrifuged at 11000 rpm for 5 min at 25°C. The supernatants were collected in an insert vial and 10 *μ*l was injected into the analytical column.

Analytical Method. The LC-MS/MS analysis was carried out using an HPLC Agilent 1260 system consisting of a model 1260 Bin solvent delivery unit, a 1260 Hip on-line degasser, a 1260 Hip ALS autoinjector, and a 1260 TCC column oven (Agilent, United States) in combination with an API 3200 mass spectrometer (AB Sciex, Singapore).

LC Conditions. The chromatographic analysis of the sample was performed on a Fortis C18 column (125 mm x 4.0 mm i.d., 5.0 *μ*m); it was fitted with a Mightysil RP-18 GP guard column (10 mm x 4.0 mm, i.d., 5.0 *μ*m) purchased from Fortis Technologies Ltd. (United Kingdom) and the Thermo Electron Corporation (United States), respectively. The column thermostat was at a controlled temperature of 25°C. The flow rate was set at 0.5 ml/min. The elution was carried out using a mixture of methanol and 0.125% ammonia solution (18:82 v/v), filtered through a 0.2 *μ*m cellulose acetate membrane filter (Sartorius, Germany) before use. The injection volume was 10 *μ*l.

MS/MS Conditions. The mass spectrometer was operated in multiple reaction monitoring (MRM) mode and was equipped with an electrospray ionization (ESI) source. Quantification was achieved with MS/MS detection in negative ion mode. The analyte (SLB) and internal standard (NRG) were monitored at transition m/z 481.007→125.110 and 270.932→150.912, respectively. The ion spray voltage was set at −4500 V. The nebulizer gas (GS1), auxiliary gas (GS2), curtain gas (CUR), and collision gas (CAD) were set at 60, 55, 35, and 6 psi, respectively. The declustering potential (DP), entrance potential (EP), collision cell entrance, potential (CEP), collision energy (CE), and collision cell exit potential (CXP) were −50, −8.5, −18, −42, and −6 V for the SLB and −40, −10, −16, −11, and −6 V for the NRG.

The analytical method for determination of silybin in human plasma by LC-MS/MS was developed and validated according to EMA and US FDA guidance including precision, accuracy, selectivity, linearity, recovery, and stability.

### 2.4. Pharmacokinetic Parameters and Tests

The pharmacokinetic parameters were analyzed including C_max_, t_max_, AUC_0-t_, AUC_0-inf_, k_e_, t_1/2_, CL, and Vd. C_max_ and t_max_ came from the actual data. The trapezoidal rule (linear up log down) was used to calculate AUC_0-t_. AUC_0-inf_ was estimated from AUC_0-t_, the last plasma concentration (C_last_), and the elimination rate constant (k_e_). The elimination rate constant (k_e_) was calculated from −2.303 multiplied by the slope of the linear regression equation curve of the log-transformed plasma drug concentrations at the terminal log linear phase. The elimination half-life (t_1/2_) was calculated as 0.693 divided by k_e_. The equations for calculating Vd and CL were dose/(*λ*_z_.AUC_inf_obs_) and dose/AUC_inf_obs_, respectively. The pharmacokinetic parameter determination was performed using Phoenix™ Winnonlin® 6.3 computer software.

### 2.5. Statistical Analysis

The pharmacokinetic data were expressed as mean ± SD. A ninety-five percent confidence interval (95% CI) for the mean of AUC _0-t_, AUC _0-inf_, and C_max_ was reported. The data from all the volunteers participating in the entire study were used for the pharmacokinetic parameters and statistical analysis.

## 3. Results 

### 3.1. The Results

Twelve healthy Thai volunteers consisting of 6 males and 6 females took part in the study. The mean age ± SD of the volunteers was 25.8 ± 5.2 years (a range of 20–35 years). The mean weight ± SD of the volunteers was 61.4 ± 5.5 kg (a range of 51.0–71.7 kg). The mean height ± SD of the volunteers was 165.4 ± 7.6 cm (a range of 156–180 cm). The mean BMI ± SD of the volunteers was 22.4 ± 1.3 kg/m^2^ (a range of 19.8–24.1 kg/m^2^).

**Figure 1:**
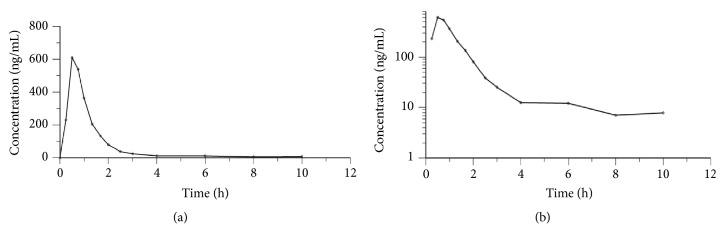
Average plasma silybin concentrations at various sampling times for all the subjects after taking a single oral dose of silymarin SMEDDS soft capsules (n = 12), (a) normal scale, (b) semilog scale.

**Figure 2:**
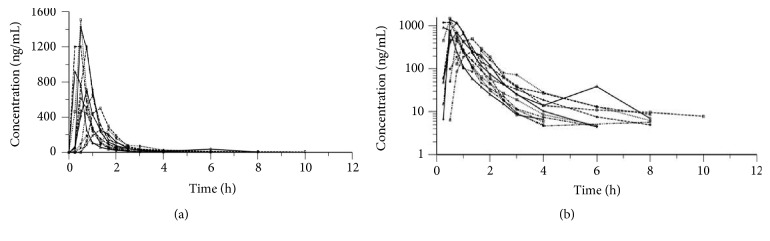
Individual plasma concentration-time curves for all the subjects after taking a single oral dose of silymarin SMEDDS soft capsules (n = 12), (a) normal scale, (b) semilog scale.

The average plasma silybin concentration of the silymarin SMEDDS soft capsules is shown in Table 1 and the concentration-time curve is presented in Figure 1. The individual volunteer plasma concentration-time curves from all the volunteers after taking silymarin SMEDDS soft capsules are drawn as spaghetti plots, as shown in Figure 2, in both a linear scale and a semilog scale. The graphs show roughly the small variation between the volunteers.

The pharmacokinetic parameters were calculated by a noncompartmental model using Phoenix Winnonlin 6.3 software. These parameters include C_max_, t_max_, AUC_0-t_, AUC_0-inf_, k_e_, t_1/2_, CL, and Vd. The pharmacokinetic parameters, i.e., t_max_, C_max_, AUC_0-t_, and AUC_0-inf_, are collated in Table 2. The mean (range) C_max_ value was 812.43 (259.47–1505.47) ng/ml and the mean (range) AUC_0-t_ value was 658.80 (268.29–1045.01) ng.h/ml. The mean (range) AUC_0-inf_ value was 676.98 (274.10–1050.96) ng.h/ml. The mean (range) t_max_ value was 0.80 (0.25–1.67) h.

The elimination phase constant (k_e_) was calculated based on the log-transformed plasma drug concentration-time curve at the terminal phase of elimination. The time points used for the linear regression analysis in the k_e_ calculation were selected automatically by the Phoenix Winnonlin 6.3 computer software, according to the “Best Fit” method. The regression analysis parameters k_e_ and t_1/2_ are presented in Table 2. The average k_e_ value was 0.5386 h^−1^. The average t_1/2_ value was 1.91 h.

A ninety-five percent confidence interval (95% CI) for the lower and upper mean for C_max_, AUC_0-t_, and AUC_0-inf_ is shown in Table 3. Ninety-five percent of the data for the mean C_max_ were within the range 536.63–1088.23 ng/mL. Ninety-five percent of the data for the means AUC_0-t_ and AUC_0-inf_ were within the ranges 489.64–827.96 ng.h/mL and 506.49–847.48 ng.h/mL, respectively.

Pharmacokinetic parameters show no difference between male and female volunteers except for t_max_ (0.54 ± 0.19 versus 1.06 ± 0.48 h) (p<0.05).

Adverse events (AEs) were followed and recorded according to the interview data and physical examination results. With regard to the safety of subjects, adverse events were observed and concomitant drug assessments were performed throughout the study. The volunteers' blood pressure, pulse rate, respiratory rate, and body temperature were within the normal range determined at their screening and during the entire study period. Their tolerance to formulations was good. There were no adverse events/serious adverse events throughout this study.

A simple, sensitive, and specific LC-MS/MS method was developed and validated for determination of silybin in human plasma. Sufficient separation of silybin peak and naringenin (internal standard) peak from other interferences was observed. The calibration curve for the analyte was linear in the range of 4–2000 ng/ml with coefficient of determination ≥ 0.99. The lower limit of quantification for silybin was 4 ng/ml. The within-run and between-run accuracy and precision were within the acceptable ranges. At room temperature, silybin was stable in plasma for at least 5 hours, and for at least 37 days at −30 ± 5 degree Celsius. The method has been found to be highly precise, accurate, robust, and suitable for the determination of silybin in plasma samples.

## 4. Discussion

The silymarin SMEDDS formulation in previous animal study has shown the enhancing bioavailability properties after oral administration [13, 14], being consistent with the present study when approximately comparing the reference capsule (equivalent to 60 mg silybin). Woo et al. [14] formulated silymarin in a SMEDDS that consisted of silymarin, glyceryl monooleate, polysorbate 20, and Transcutol®. The release rate of the silybin from the SMEDDS was 2.5 times faster than that from the reference capsule. After its oral bioavailability in rats, the relative bioavailability of the drug from the SMEDDS was 360% of the reference formulation. Wu et al. [7] formulated silymarin in a SMEDDS formulation composed of silymarin, Tween 80, ethyl alcohol, and ethyl linoleate. The pharmacokinetic and relative bioavailability of silymarin in the form of a SMEDDS was determined in rabbits, along with suspension and solution formulation. The relative bioavailability of the SMEDDS formulation was clearly enhanced approximately twofold and 49-fold that of the solution and suspension formulation, respectively. Li et al. [13] optimized the solubility of silymarin by incorporating ethyl linoleate, Cremophor EL, and ethyl alcohol into the SMEDDS formulation. According to a bioavailability study on dogs, a clearly faster release (2.2 times) of silybin in a SMEDDS formulation capsule than in a reference capsule was reported. The pharmacokinetic data of silybin following a single-dose oral administration of silymarin SMEDDS soft capsules in this study were compared indirectly with the published data of conventional capsules, as presented in Table 4; both studies were on healthy human volunteers with similar characteristics [15, 16]. According to those studies, the t_max_ after the oral administration of silybin in a SMEDDS capsule was approximately 2 times faster than in a conventional capsule. Moreover, the C_max_ and AUC were approximately 4.9–5.9 and 1.7–2.5 times higher than a conventional capsule. In addition, if compared to a different commercial preparation of a powdered milk thistle extract capsule [17, 18], the C_max_ and AUC of silybin from the silymarin SMEDDS soft capsules were approximately 34–62 and 9.5–26.3 times higher, respectively, than from the powdered milk thistle extract capsule. However, there were no previous reports about pharmacokinetic, bioavailability, and bioequivalence study of silymarin in the form of SMEDDS; thus pharmacokinetic data cannot be compared to the present study.

The elimination half-life of silybin in humans was previously reported as being 1.3–7.8 h after the administration of various forms of silymarin formulation and was shown to be within the same range in this study (0.73–7.50 h).

The above data indicated that after oral administration of silybin in a SMEDDS formulation, the absorption of the silybin was faster, and the extent of the absorption was enhanced. However, further study with the same group of volunteers using a noninferior test is recommended for a more appropriate comparison. It would be more beneficial to conduct study on hepatic disease patients who might have difference in pharmacokinetic parameters.

## 5. Conclusions

After a single oral dose of silymarin SMEDDS soft capsules, the mean (range) C_max_ value was 812.43 (259.47–1505.47) ng/ml and the average AUC_0-t_ value was 658.80 (268.29–1045.01) ng.h/ml. The average AUC_0-inf_ value was 676.98 (274.10–1050.96) ng.h/ml. The mean (range) t_max_ value was 0.80 (0.25–1.67) h. It showed a fast absorption (t_max_ < 1 h) and high oral bioavailability (C_max_: 812.43 ng/ml, AUC_0-t_: 658.80 ng.h/ml). The subjects tolerated the study well, as no clinically significant or serious adverse drug reactions were noticed.

## Figures and Tables

**Table 1:** Average plasma silybin concentrations at various sampling times for all the volunteers after taking a single oral dose of silymarin SMEDDS soft capsules (n = 12).

**Time (h)**	**Plasma silybin concentration (ng/ml)**
0.0	0.00 ± 0.00
0.25	231.51 ± 413.00
0.50	610.71 ± 542.27
0.75	539.20 ± 368.87
10	364.05 ± 215.60
1.33	205.75 ± 126.04
1.67	134.66 ± 86.29
2.0	80.52 ± 53.85
2.5	38.86 ± 22.13
3.0	25.28 ± 18.79
4.0	12.60 ± 8.27
6.0	12.21 ± 11.38
8.0	7.09 + 1.82
10.0	7.88
12.0	-

**Table 2:** Pharmacokinetic parameters for the volunteers after taking a single oral dose of silymarin SMEDDS soft capsules (n=12).

Pharmacokinetic parameters (unit)	Arithmetic mean ± SD	Range
C_max_ (ng/ml)	812.43 ± 434.07	259.47–1505.49
AUC_0-t_ (ng.h/ml)	658.80 ± 266.23	268.29–1045.01
AUC_0-inf_ (ng.h/ml)	676.98 ± 268.34	274.10–1050.96
t_max_ (h)	0.80 ± 0.44	0.25–1.67
k_e_ (h^−1^)	0.5386 ± 0.2634	0.0924–0.9508
T_1/2_ (h)	1.91 ± 1.85	0.73–7.50
CL (L.h)	104.50 ± 47.51	57.09–218.90
Vd (L)	263.95 ± 229.63	75.86–909.46

C_max_: maximum observed plasma concentration; AUC_0-t_: area under the plasma concentration versus time curve up to the last; AUC_0-inf_: area under the plasma concentration versus time curve with the concentration extrapolated based on the elimination rate constant; t_max_: time to C_max_; k_e_: elimination rate constant; T_1/2_: elimination half-life; CL: clearance; Vd: volume of distribution.

**Table 3:** 95% confidence interval (lower and upper mean) of the pharmacokinetic parameters of silybin after the oral administration of silymarin SMEDDS soft capsules.

Parameters	95% Confidence Interval (lower and upper mean)
C_max_ (ng/mL)	536.63–1088.23
AUC_0-t_ (ng.h/mL)	489.64–827.96
AUC_0-inf_ (ng.h/mL)	506.49–847.48

C_max_: maximum observed plasma concentration; AUC_0-t_: area under the plasma concentration versus time curve up to the last; AUC_0-inf_: area under the plasma concentration versus time curve with the concentration extrapolated based on the elimination rate constant.

**Table 4:** Pharmacokinetic parameters of silybin after the oral administration ofsilymarin SMEDDS soft capsules compared with conventional capsules from the published study.

**Pharmacokinetics** **Parameters**	**Silybin (mean ± SD)**		
	**Silymarin SMEDDS soft capsules**	**Published study**	
		**Conventional capsule** ^**∗**^	**Conventional** ^**∗∗**^
t_max_ (h)^#^	0.80 (0.25-1.67)	1.50 (0.50-8.0)^#^	1.5
C_max_ (/ngml)	812.43 ± 434.07	137.40 ± 51.83	167.3
AUC_0-t_ (ng.h/ml)	658.80 ± 266.23	294.80 ± 95.58	N/A
AUC_0-inf_ (ng.h/ml)	676.98 ± 268.34	299.10 ± 96.00	406.5

*Note*. ^*∗*^ Published study conducted by Zhu and coworkers in 2013 [15].

^*∗∗*^ Published study conducted by Brinda and coworkers in 2012 [16].

^#^ Median (range).

C_max_: maximum observed plasma concentration; AUC_0-t_: area under the plasma concentration versus time curve up to the last; AUC_0-inf_: area under the plasma concentration versus time curve with the concentration extrapolated based on the elimination rate constant; t_max_: time to C_max_; N/A: not available.

## Data Availability

Data underlying this research will be sent by email to the publisher and are available from the corresponding author upon request.

## References

[1] Fraschini F., Demartini G., Esposti D. (2002). Pharmacology of silymarin. *Clinical Drug Investigation*.

[2] Radko L., Cybulski W. (2007). Application of silymarin in human and animal medicine. *Journal of Pre-Clinical and Clinical Research*.

[3] Wu J.-W., Lin L.-C., Tsai T.-H. (2009). Drug-drug interactions of silymarin on the perspective of pharmacokinetics. *Journal of Ethnopharmacology*.

[4] Mayer K. É., Myers R. P., Lee S. S. (2005). Silymarin treatment of viral hepatitis: a systematic review. *Journal of Viral Hepatitis*.

[5] Dixit N., Baboota S., Kohli K., Ahmad S., Ali J. (2007). Silymarin: a review of pharmacological aspects and bioavailability enhancement approaches. *Indian Journal of Pharmacology*.

[6] Javed S., Kohli K., Ali M. (2011). Reassessing bioavailability of silymarin. *Alternative Medicine Review*.

[7] Wu W., Wang Y., Que L. (2006). Enhanced bioavailability of silymarin by self-microemulsifying drug delivery system. *European Journal of Pharmaceutics and Biopharmaceutics*.

[8] Chu Y., Li W., Li Z. W. (2011). Simultaneous determination of silybin A and silybin B in rat plasma and pharmacokinetic study. *Chinese Herbal Medicine*.

[9] Li W., Han J., Li Z., Li X., Zhou S., Liu C. (2008). Preparative chromatographic purification of diastereomers of silybin and their quantification in human plasma by liquid chromatography-tandem mass spectrometry. *Journal of Chromatography B*.

[10] Kim Y. C., Kim E. J., Lee E. D. (2003). Comparative bioavailability of silibinin in healthy male volunteers. *International Journal of Clinical Pharmacology and Therapeutics*.

[11] Hussein A., El-Menshawe S., Afouna M. (2012). Enhancement of the in-vitro dissolution and in-vivo oral bioavailability of silmarin from liquid-filled hard gelatin capsules of semisolid dispersion using Gelucire 44/14 as carrier. *Pharmacie*.

[12] Chang L.-W., Hou M.-L., Tsai T.-H. (2014). Silymarin in liposomes and ethosomes: Pharmacokinetics and tissue distribution in free-moving rats by high-performance liquid chromatography-tandem mass spectrometry. *Journal of Agricultural and Food Chemistry*.

[13] Li X., Yuan Q., Huang Y., Zhou Y., Liu Y. (2010). Development of silymarin self-microemulsifying drug delivery system with enhanced oral bioavailability. *AAPS PharmSciTech*.

[14] Jong S. W., Kim T.-S., Park J.-H., Chi S.-C. (2007). Formulation and biopharmaceutical evaluation of silymarin using SMEDDS. *Archives of Pharmacal Research*.

[15] Zhu H.-J., Brinda B. J., Chavin K. D., Bernstein H. J., Patrick K. S., Markowitz J. S. (2013). An assessment of pharmacokinetics and antioxidant activity of free silymarin flavonolignans in healthy volunteers: a dose escalation study. *Drug Metabolism and Disposition*.

[16] Brinda B. J., Zhu H.-J., Markowitz J. S. (2012). A sensitive LC-MS/MS assay for the simultaneous analysis of the major active components of silymarin in human plasma. *Journal of Chromatography B*.

[17] Wen Z., Dumas T. E., Schrieber S. J., Hawke R. L., Fried M. W., Smith P. C. (2007). Pharmacokinetic and metabolic profile of free, conjugated, and total silymarin flavonolignans in human plasma after oral administration of milk thistle extract. *Drug Metabolism and Disposition*.

[18] Schrieber S. J., Wen Z., Vourvahis M. (2008). The pharmacokinetics of silymarin is altered in patients with hepatitis C virus and nonalcoholic fatty liver disease and correlates with plasma caspase-3/7 activity. *Drug Metabolism and Disposition*.

